# Guillain-Barré Syndrome presenting with bilateral facial nerve paralysis: a case report

**DOI:** 10.1186/1757-1626-1-379

**Published:** 2008-12-08

**Authors:** Ram Prakash Narayanan, Nirmal James, Kannan Ramachandran, Mario J Jaramillo

**Affiliations:** 1 Department of Diabetes and Endocrinology, University Hospital of Aintree, Lower Lane, Liverpool L9 7AL, UK; 2 Department of Anaesthesia, University Hospital of Wales, Heath Park, Cardiff CF14 4XW, UK; 3 Department of ENT Surgery, West Wales General Hospital, Carmarthen SA31 2AF, UK

## Abstract

Bilateral paralysis of the facial nerve is a relatively rare presentation and often indicates a serious underlying medical condition. Guillain-Barré syndrome needs to be considered, among others in the differential diagnoses of such presentation. We present here the case of a 35 year old female who presented with bilateral facial nerve paralysis due to the Guillain-Barré syndrome.

## Introduction

Unilateral facial paralysis is a common clinical entity. Majority of these cases are due to idiopathic or Bell's palsy. Bilateral facial paralysis, unlike its unilateral counterpart is an extremely rare presentation. An aetiological factor is often demonstrable [1]. Majority of these cases are due to serious underlying medical conditions and may need emergency medical treatment. Common causes for bilateral facial palsy include Lyme disease, Guillain-Barre syndrome, Leukaemia, Sarcoidosis, Infectious Mononucleosis and trauma. Only 20% of these cases are due to Idiopathic or Bell's palsy where no evidence of systemic or local disease can be found [2]. We present a case report of bilateral facial paralysis due to Guillain-Barré syndrome which has been successfully managed.

## Case Presentation

A 35-year-old Caucasian female school teacher initially presented with a one day history of left sided facial weakness. Two weeks earlier she was treated by her general practitioner with a course of oral amoxicillin for sore throat. There was no other significant past medical history and she was on no regular medications. The patient was a non-smoker and did not consume alcohol. An otolaryngological examination was unremarkable but for incomplete left lower motor neuron type of facial palsy (House & Brackman Grade III). A provisional diagnosis of Idiopathic Facial Paralysis (Bell's Palsy) was made and the patient was discharged home after reassurance and on no specific medications.

Two days later she returned with paralysis now involving both sides of the face. While she did give a history of 'pins and needles' on both hands and feet, and stiff shoulders, she denied any numbness over the sites. She denied any history of trauma, rashes, travel abroad or exposure to tick bites. She was aware of altered taste sensation. Examination revealed bilateral complete lower motor neuron type of facial palsy (House & Brackman Grade VI). All other cranial nerves were intact and there was no evidence of sensory deficits elsewhere. Lower limb power was 4/5 across all muscle groups, while power in the upper limbs was normal. By the tenth day, she developed loss of ankle reflexes bilaterally. Paradoxically, at this time, the biceps, triceps and jaw reflexes were brisk. Plantar reflexes were flexor throughout. There was no bladder or bowel involvement at any point. Fundoscopy was normal.

Blood tests for full blood counts, urea and electrolytes, serum angiotensin converting enzyme (ACE) and the vasculitic screen were within normal limits. Blood culture was negative. ESR was 20 mm/1^st ^hr. Chest radiograph was clear. No paraproteins were detected on serum protein electrophoresis. Pure tone audiometry revealed normal hearing thresholds on both sides and Tympanometry showed normal middle ear mechanism on both sides. Magnetic Resonance Imaging scan of the head was normal.

Nerve conduction studies showed prolonged F-wave from right abductor digiti minimi (ADM) and prolonged distal motor latency to right abductor pollicis brevis (APB) suggestive of an early Acute Inflammatory Demyelinating Polyneuropathy (AIDP). Good response was seen to nerve conduction tests on facial muscles. Lumbar puncture was then performed revealing an albumin-cytological dissociation (CSF protein of 0.97 g/L and a white cell count of zero). CSF glucose was 4.7 mmol/l (plasma glucose 8.0 mmol/l). With the characteristic protein-cell count differentiation, a diagnosis of the Guillain-Barre Syndrome (Acute Inflammatory Demyelinating Polyneuropathy) was made. Pulmonary function assessments were within normal limits. CSF cultures, stool cultures (including for campylobacter), throat swabs for streptococci and viral serology for Varicella-Zoster and herpes simplex were all negative. Similarly, the Monospot test for Infectious Mononucleosis was negative. Anti-Nuclear antibodies (ANA), Anti-Nuclear Cytoplasmic antibodies (ANCA) and Anti-Double Stranded DNA (Anti dsDNA) studies were also negative.

**Figure 1:**
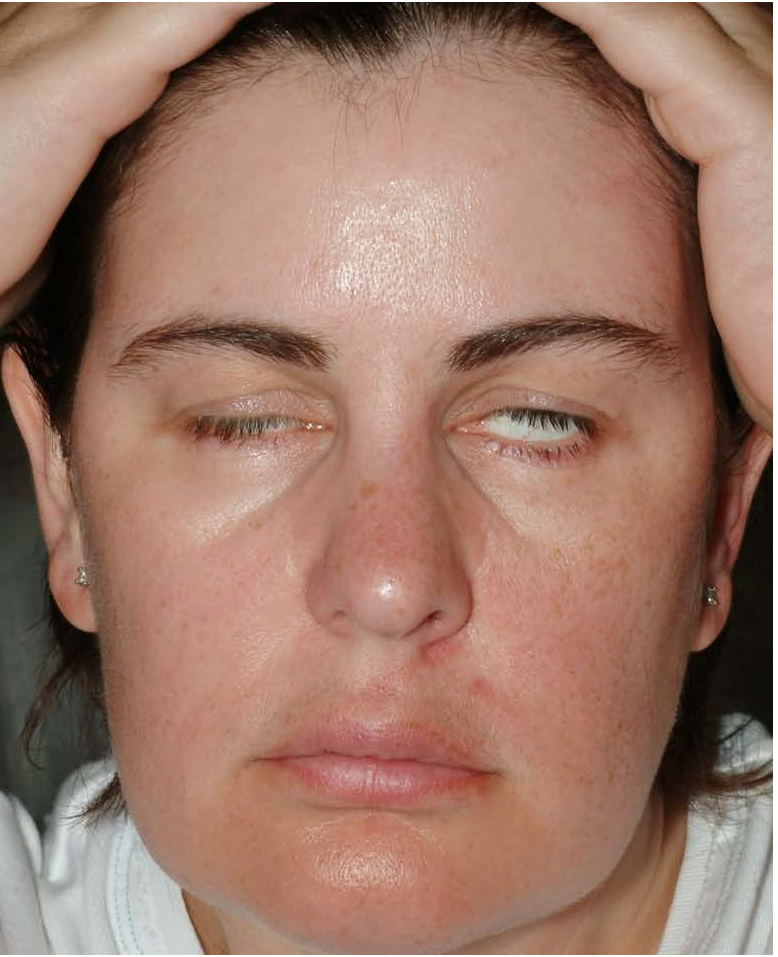
Day 10: Partial recovery of the right side of face after 3 days of intravenous immunoglobulins.

She was commenced on a course of oral steroids on day 3 (60 mg oral prednisolone) – the day of first presentation with bilateral palsy and this was reduced gradually to be stopped by day 18. Appropriate eye protection measures were taken. After confirmation of AIDP, she was initiated on a five day course of 30 g of intravenous (iv) immunoglobulin infusions (octagam^®^) from day 8 (Fig 1 shows the patient after 3 days of iv immunoglobulin). She was then transferred to the regional neurology unit where a repeat lumbar puncture was done confirming AIDP once again. Tests were negative for Lyme disease, Herpes simplex, Borrelia burgdorferi and Oligoclonal bands.

Her facial muscle function gradually improved, beginning with the right side. She was discharged on day 16, by then she was feeling significantly better and power and reflexes in both the upper and lower limbs were normal. Advice was given to continue eye protection and return if there was any suggestion of worsening weakness or respiratory distress. She progressively improved and facial weakness had almost completely resolved on the right side by day 30. Her ankle reflexes returned bilaterally. While both sides the facial nerves had recovered significantly (House & Brackman Grade II), the residual deficit was more on the left side.

## Discussion

Bilateral facial nerve palsy has an incidence of only 1 per 5 million populations per year [3] It may be the presenting feature of a potentially life threatening illness, hence care must be taken to exclude potential metabolic, infectious, vasculitic, traumatic [1], immunological (eg. multiple sclerosis) and neoplastic causes before diagnosing a bilateral Idiopathic or Bell's palsy [4].

Lyme's disease is a common cause of facial palsy in endemic areas but our patient had no history of exposure to ticks or recent travel abroad. There was no evidence of erythema chronicum migrans or the characteristic rash. In view of her age and atypical findings, she was investigated for multiple sclerosis, but the magnetic resonance imaging of the head was normal and cerebrospinal fluid analysis was negative for oligoclonal bands. Herpes viruses and infectious mononucleosis may also affect the facial nerve but the screen for herpes simplex and varicella-zoster viruses were negative.

Sarcoidosis [5], systemic lupus erythrematosus (SLE) [6] and Polyarteritis Nodosa (PAN) are other diseases associated with facial diplegia, but with a low ESR and a negative auto-antibody screen they were considered less probable. The patient's presentation and normal MR brain imaging made CNS leukaemia, lymphoma and benign intracranial hypertension unlikely. Other causes listed in the literature include amyloidosis, syphilis, poliomyelitis, tuberculosis and porphyria [7] but in view of their rarity in our patient's circumstances these possibilities were not explored further. Wegener's granulomatosis involving middle ears on both sides causing bilateral facial paralysis has also been reported [8].

Guillain-Barré syndrome, also known as an Acute Inflammatory Demyelinating Polyneuropathy (AIDP) is an acute demyelinating polyradiculopathy of uncertain aetiology which may present with facial nerve involvement in 27–50% of cases, often bilaterally [9]. In many cases other cranial nerves may also be involved, with the possibilities of coexistent dysphagia and dysarthria. A history of a preceding viral infection is seen in most cases. Facial palsy usually follows limb weakness [10]. Our patient presented rather unusually in that her facial nerve paralysis preceded any significant areflexia in the peripheral limbs, the so called 'descending variant' and loss was restricted to the ankle reflexes. Diagnosis was made by cerebrospinal fluid analysis revealing a raised protein content in the absence of an increased cell count. Presenting features are variable and may include significant respiratory muscle paralysis, in which case invasive ventilation may be needed. Hence, early and regular pulmonary function assessments are recommended in all cases. Treatment is usually supportive, with immunoglobulin infusions or plasmapheresis in appropriate cases. Prognosis is generally good with the above measures [9].

## Conclusion

Simultaneous presentation of bilateral facial palsy is very uncommon. The differential diagnosis of these should include Acute Inflammatory Demyelinating Polyneuropathy (Guillain-Barré syndrome). Raised protein content in the absence of increased cell count at CSF analysis confirms the clinical diagnosis. Management may include ventilatory support, immunoglobulin infusion or plasmapheresis. The prognosis is good in the majority of treated cases.

## Patient's perspective

The symptoms began with a sore throat and an ulcer on the side of the tongue. After approximately 7 days these symptoms disappeared and it was not until five days later that the pins and needles and facial palsy began. Recovery on the left side was slow with some residual weakness 2 years later. Throughout the first year of recovery I had an extreme feeling of lethargy and anxiety regarding the degree of facial recovery. Intensive physiotherapy and reflexology aided this recovery.

## Consent

Written informed consent was obtained from the patient for publication of this case report and accompanying images. A copy of the written consent is available for review by the Editor-in-Chief of this journal.

## Competing interests

The authors declare that they have no competing interests.

## Authors' contributions

All the authors were involved in the patient's care. RPN and NJ were involved in the initial care of the patient and RPN first raised the possibility of GBS. KR organized the relevant investigations and MJJ was the consultant who guided the overall management. All authors read and approved this final manuscript.

## References

[B1] Hartley C, Mendelow AD (1993). Post traumatic bilateral facial palsy. J Laryngol Otol.

[B2] Stahl N, Ferit T (1989). Recurrent bilateral peripheral facial palsy. J Laryngol Otol.

[B3] Teller DC, Murphy TP (1992). Bilateral Facial Paralysis: A case presentation and literature review. The Journal of Otolaryngology.

[B4] Cwach H, Landis J, Freeman JW (1997). Bilateral Seventh Nerve Palsy: A report of Two Cases and a Review. S D J Med.

[B5] James DG (1997). Differential diagnosis of facial nerve palsy. Sarcoidosis Vasc Diffuse Lung Dis.

[B6] Blaustein DA, Blaustein SA (1998). Anti nuclear antibody negative systemic lupus erythematosus presenting as bilateral facial paralysis. Journal of Rheumatology.

[B7] Price T (2002). Bilateral simultaneous facial nerve palsy. J Laryngol Otol.

[B8] Nikolaou AC, Vlachtsis KC, Daniilidis MA, Petridis DG, Daniilidis IC (2001). Wegener's genulomatosis presenting with bilateral facial nerve palsy. European Archives of Oto-Rhino-Laryngology.

[B9] Kilic R, Ozdek A, Felek S, Safak MA, Samim E (2003). A case presentation of Bilateral Simultaneous Bell's Palsy. Americal Journal of Otolaryngology.

[B10] Keane JR (1994). Bilateral seventh nerve palsy: Analysis of 43 cases and review of the literature. Neurology.

